# Caveolin-1 regulates cancer cell metabolism via scavenging Nrf2 and suppressing MnSOD-driven glycolysis

**DOI:** 10.18632/oncotarget.5687

**Published:** 2015-10-26

**Authors:** Peter C. Hart, Bianca A. Ratti, Mao Mao, Kristine Ansenberger-Fricano, Ayesha N. Shajahan-Haq, Angela L. Tyner, Richard D. Minshall, Marcelo G. Bonini

**Affiliations:** ^1^ Department of Medicine, University of Illinois College of Medicine at Chicago, Chicago, IL, USA; ^2^ Department of Pathology, University of Illinois College of Medicine at Chicago, Chicago, IL, USA; ^3^ Department of Biochemistry and Molecular Genetics, University of Illinois College of Medicine at Chicago, Chicago, IL, USA; ^4^ Department of Pharmacology, University of Illinois College of Medicine at Chicago, Chicago, IL, USA; ^5^ Department of Anesthesiology, University of Illinois College of Medicine at Chicago, Chicago, IL, USA; ^6^ Department of Oncology, Georgetown University, Medical Center, Washington, D.C., USA; ^7^ Programa de Biociencias Aplicadas a Farmacia (PBF) Universidade Estadual de Maringa, Maringa, PR, Brazil

**Keywords:** caveolin-1, MnSOD, Nrf2, breast cancer, tumor progression

## Abstract

Aerobic glycolysis is an indispensable component of aggressive cancer cell metabolism. It also distinguishes cancer cells from most healthy cell types in the body. Particularly for this reason, targeting the metabolism to improve treatment outcomes has long been perceived as a potentially valuable strategy. In practice, however, our limited knowledge of why and how metabolic reprogramming occurs has prevented progress towards therapeutic interventions that exploit the metabolic peculiarities of tumors. We recently described that in breast cancer, MnSOD upregulation is both necessary and sufficient to activate glycolysis. Here, we focused on determining the molecular mechanisms of MnSOD upregulation. We found that Caveolin-1 (Cav-1) is a central component of this mechanism due to its suppressive effects of NF-E2-related factor 2 (Nrf2), a transcription factor upstream of MnSOD. In transformed MCF10A(Er/Src) cells, Cav-1 loss preceded the activation of Nrf2 and its induction of MnSOD expression. Consistently, with previous observations, MnSOD expression secondary to Nrf2 activation led to an increase in the glycolytic rate dependent on mtH_2_O_2_ production and the activation of AMPK. Moreover, rescue of Cav-1 expression in a breast cancer cell line (MCF7) suppressed Nrf2 and reduced MnSOD expression. Experimental data were reinforced by epidemiologic nested case-control studies showing that Cav-1 and MnSOD are inversely expressed in cases of invasive ductal carcinoma, with low Cav-1 and high MnSOD expression being associated with lower 5-year survival rates and molecular subtypes with poorest prognosis.

## INTRODUCTION

Caveolin-1 (Cav-1) is a scaffold protein primarily located at the lipid raft domains in the cellular plasma membrane. The roles of Cav-1 as a facilitator of endocytosis, transcytosis, and trafficking of cholesterol have long been acknowledged [1–4]. More recently, however, Cav-1 function as an active regulator of cell signaling has become better appreciated. By defining niches where signals are generated at restricted microdomains [3, 5, 6] or directly regulating the activity of nuclear factors [7–9] and receptors [3, 5], Cav-1 forms a molecular platform modulating a variety of signaling programs that regulate cellular responses to environmental cues [6, 10]. Noteworthy, Cav-1 expression has been reported to vary in response to toxic exposures [11–13], during cellular differentiation and migration [14, 15] and under conditions of elevated reactive oxygen species (ROS) and reactive nitrogen species (RNS) production [16–18]. Data from other groups have indicated that loss of Cav-1 acutely accentuates the production of H_2_O_2_ and promotes oxidative stress [19, 20]. When persistent, suppression of Cav-1 expression appears to promote the emergence of pathogenic phenotypes [16, 19, 21] characterized by a stronger reliance on glycolytic metabolism [19, 22] and active cellular proliferation [23–25]. It is, however, unclear how variations in Cav-1 expression translate into robust changes in cellular metabolism, particularly in the case of cancer cells.

Here, we demonstrate that the reconstitution of Cav-1 expression in invasive MCF7 breast ductal carcinoma cells that are normally deficient in Cav-1 suppresses glycolysis and enhances mitochondria-dependent ATP production. These effects were largely dependent on the suppression of Nrf2-driven upregulation of MnSOD which, as previously shown [26], is required and sufficient for the glycolytic switch. Moreover, we found strong correlations between tumor grade, tumor stage, and 5-year breast cancer survival with Cav-1 expression level, with low Cav-1 expression being associated with higher tumor grade and lower 5-year survival. These observations indicate that perhaps low levels of Cav-1 expression distinguish a subgroup of aggressive breast cancers with strong reliance on glycolytic metabolism.

## RESULTS

### Cav-1 and MnSOD are inversely expressed in human breast cancer and predict risk of aggressive phenotypes

**Figure 1:**
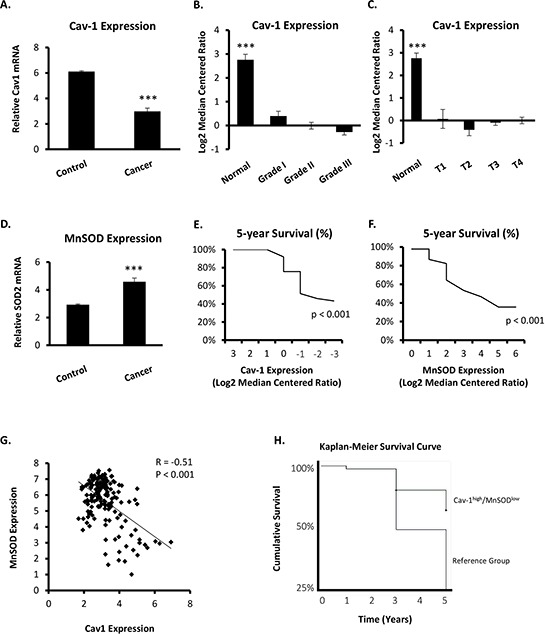
Cav-1 and MnSOD mRNA levels are inversely expressed in human breast cancer patients Nested case-control studies were performed on data previously obtained by Sorlie [27](Figure 1A–1F) and Curtis [28](Figure 1G and 1H). Cav-1 and MnSOD mRNA expression obtained from a primary site is expressed as log2 median centered ratio for case (invasive ductal carcinoma) and control (normal breast tissue) subjects. **A.** Cav-1 mRNA expression between controls and all cases with IDC (*N* = 116). **B.** Cav-1 mRNA expression stratified by grade (*N* = 116). **C.** Cav-1 mRNA expression stratified by tumor size **(T)** (*N* = 116). **D.** MnSOD mRNA expression between controls and all cases with IDC (*N* = 116). **E.** Cohort is stratified using Cav-1 mRNA expression set as the continuous variable to determine Cav-1-dependent survival curve (*N* = 116). Comparison between highest and lowest Cav-1 mRNA expression demonstrated a significant reduction in survival in patients with lowest Cav-1, as assessed by Pearson's Chi-Square Test. **F.** Cohort is stratified using MnSOD mRNA expression set as the continuous variable to determine MnSOD-dependent survival curve (*N* = 116). In contrast to Figure 1E, analysis between lowest and highest MnSOD mRNA expression showed a marked increase in mortality in patients with highest MnSOD, as determined by Pearson's Chi-Square Test. **G.** Correlation plot of Cav-1 and MnSOD expression demonstrating a moderate inverse/negative correlation of Cav-1 and MnSOD in patients with aggressive invasive ductal carcinoma (e.g., medullary carcinoma). **H.** Kaplan-Meier survival estimate curve in patients stratified by high Cav-1 (quartile 4) paired with low MnSOD (quartile 1) compared to the reference group using data obtained previously by Kao [29]. (One-way ANOVA with post-hoc two-sided *t*-test, *** = *p* < 0.001; Student's two-sided *t*-test, *** = *p* 0.001;).

A nested case-control study of existing data by Sorlie *et al*. (Figure 1A–1F, [27]) and Curtis *et al*. (Figure 1G and 1H, [28]) showed that Cav-1 mRNA is markedly reduced in cancerous breast tissue as compared to healthy controls. Cav-1 expression was markedly reduced in invasive ductal carcinoma (Figure 1A–1C) and observed to progressively correlate in a grade- and stage-dependent manner (Figure 1B and 1C, respectively). Noteworthy, robust suppression of Cav-1 gene expression was observed between the first stage of malignancy (grade 1 and T1) and normal tissue. Within the examined cohort, it was also observed that MnSOD mRNA expression was significantly increased in invasive breast cancer (Figure 1D). Importantly, stratification of this population by either decreasing Cav-1 or increasing MnSOD expression showed that lower Cav-1 (and higher MnSOD) indicated a significant increase in mortality (Figure 1E and 1F, respectively). Interestingly, Cav-1 and MnSOD had a moderate negative correlation (Pearson's *R* = −0.51, *p* < 0.001) in patients with invasive breast cancer (Figure 1G), and stratification of this cohort by low Cav-1 and high MnSOD expression in invasive ductal carcinoma indicated increased mortality (odds ratio, OR = 1.576, 95% CI 1.076 – 2.307, *p* < 0.05) and conferred risk of aggressive disease (OR = 2.099, 95% CI 1.321 – 3.333, *p* < 0.005), as shown in Table 1. The analysis of available data also indicated that the expression level of Cav-1 and MnSOD in human breast cancer has weak negative prognostic value independently, but in combination, the Cav-1^low^/MnSOD^high^ signature is strongly correlated with more aggressive forms of the disease. In contrast, stratification of this cohort by high Cav-1 and low MnSOD conferred a two-fold lower risk of death from the disease (OR = 0.545, 95% CI 0.354 – 0.839, *p* < 0.01), and sufficiently discriminated between healthy subjects and patients with invasive ductal carcinoma (OR = 0.203, 95% CI 0.127 – 0.324, *p* < 0.01, Table 2), together indicating that this molecular fingerprint may have prognostic value for risk stratification. This notion was reinforced by a separate study from Kao *et al.* [29] showing that the Cav-1^low^/MnSOD^high^ phenotype had a higher mean survival time of 4.6 compared to 3.9 years in the reference group (Figure 1H). Taken together, the relationship between Cav-1 and MnSOD appears to have predictive value indicating more invasive forms of breast cancer.

**Table 1:** Stratification of patients with invasive ductal carcinoma by Cav-1^low^ and MnSOD^high^

	*Basal Phenotype*	*Mortality*
***Reference Group (%)***	181 (1.61)	669 (5.86)
***Cases [Cav-1^low^/MnSOD^high^] (%)***	26 (22)	61 (53)
***Odds Ratio***	2.099	1.576
***CI 95%***	1.321 – 3.333	1.076 – 2.307
***p value***	*p* < 0.05	*p* < 0.05

**Table 2:** Stratification of patients with invasive ductal carcinoma by Cav-1^high^ and MnSOD^low^

	*IDC*	*Mortality*
***Reference Group (%)***	1480 (92)	699 (43)
***Cases [Cav-1^high^/MnSOD^low^] (%)***	75 (72)	31 (1.9)
***Odds Ratio***	0.203	0.545
***CI 95%***	0.127 – 0.324	0.354 – 0.839
***p value***	*p* < 0.01	*p* < 0.01

### The expression of Cav-1 and Nrf2 are inversely associated in human breast cancer

**Figure 2:**
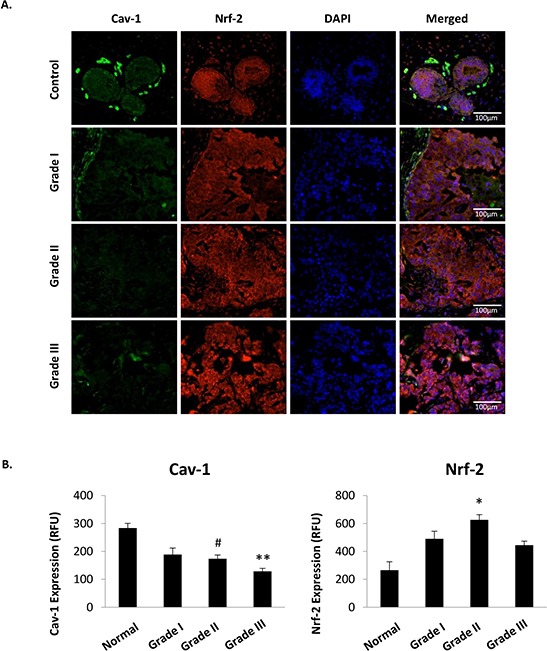
Cav-1 and Nrf2 protein expression are inversely associated in human breast cancer tissue **A.** Cav-1 (green) and Nrf2 (red) protein expression in invasive ductal carcinoma tissue obtained from TMA-1005 (Protein Biotechnologies). **B.** Representative images were selected by identifying images with closest measurements to that of the mean derived for each grade. (One-way ANOVA with post-hoc two-sided *t*-test, ** = *p* < 0.005, *** = *p* < 0.001).

Findings of epidemiologic associations between levels of Cav-1 and MnSOD expression and breast cancer outcome led us to perform a set of prospective studies using a tissue microarray containing 36 breast cancer cases and 12 normal tissue controls stratified by histologic grade and clinical stage (TMA-1005, Protein Biotechnologies, Ramona, CA) to detect correlations between Cav-1/MnSOD phenotypes and clinical grade and stage. Figure 2A/2B summarizes our findings that Cav-1 expression is progressively reduced in ductal epithelial cells by increasing histologic grade and clinical stage. Cav-1 was previously shown to bind and suppress NF-E2-related factor 2 (Nrf2), a nuclear factor upstream of MnSOD expression [8, 30]. Consistent with these findings by other groups, we observed a clear inverse relationship between Cav-1 and Nrf2 expression especially in higher grade (II-III) tumors.

### Cav-1 expression in MCF7 breast cancer epithelial cells represses Nrf2 and MnSOD

**Figure 3:**
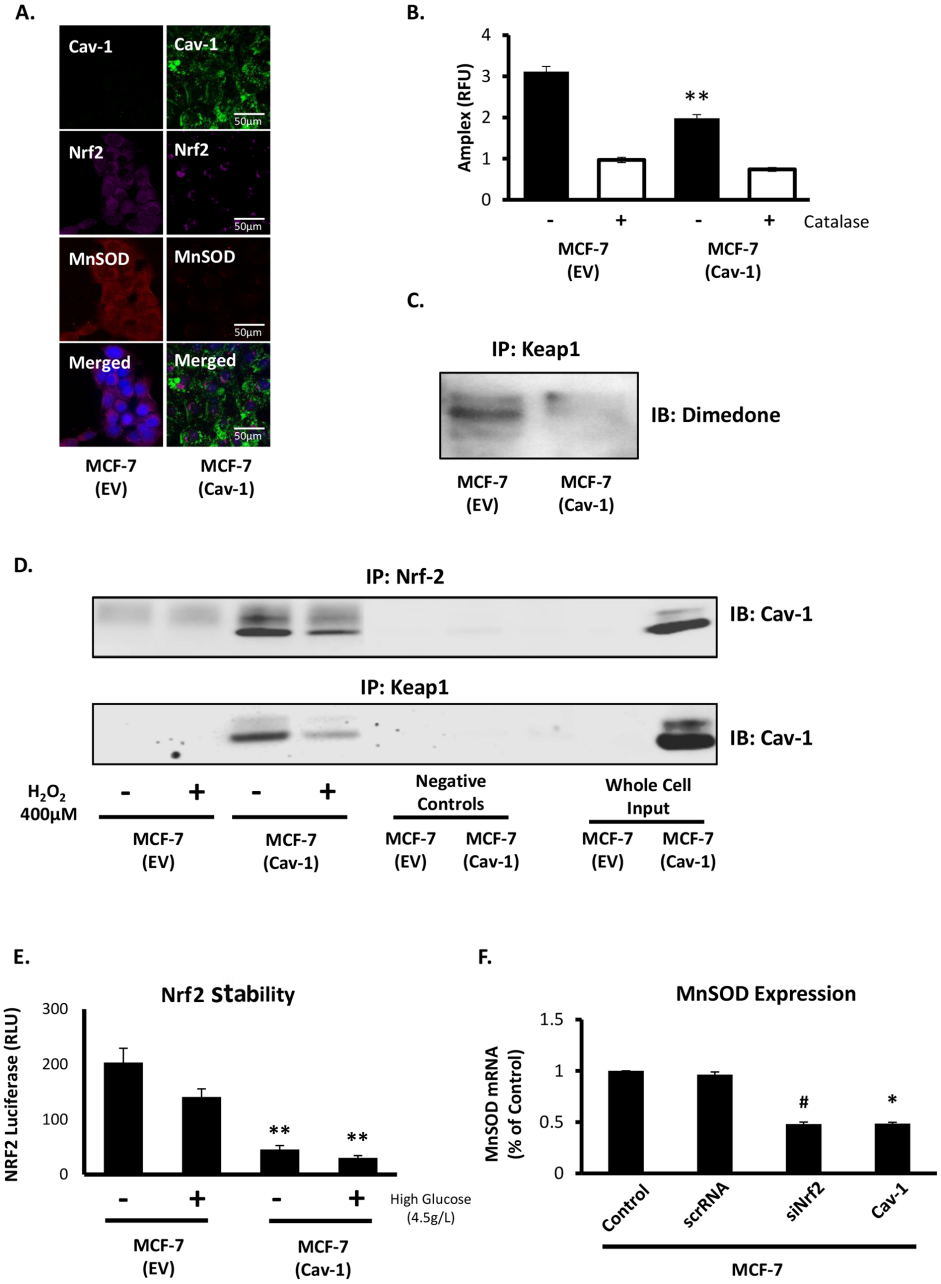
Cav-1 prevents MnSOD upregulation by repression of Nrf2 **A.** Cav-1 (green) transfected MCF7 cells demonstrate a reduction of Nrf2 (purple) and MnSOD (red). **B.** H_2_O_2_ production in MCF7 cells expressing Cav-1 is reduced when compared to MCF7 cells devoid of Cav-1. H_2_O_2_ was measured as described in *methods*. **C.** Cysteine sulfenic acid formation in MCF7 empty vector and Cav-1 expression cells using dimedone in combination with immunoprecipitation of Keap1 shows a reduction in Keap1 thiol oxidation in Cav-1 expressing MCF7 cells. **D.** Immunoprecipitation of Nrf2 (top) and Keap1 (bottom) demonstrates direct interactions of both proteins to Cav-1. Cells were treated with 400 μM H_2_O_2_ for 1 h prior to preparation of lysates for immunoprecipitation as described in *methods*. **E.** Nrf-2 transcriptional activity was indirectly determined using NanoLuc Nrf2-stability luciferase reporter assay in MCF7 cells with and without Cav-1 stable expression in both DMEM:F12 and DMEM high glucose (4.5g/L) conditions. **F.** MnSOD mRNA expression following Cav-1 ectopic expression or silencing of Nrf2 in MCF7 cells shows a similar reduction in MnSOD mRNA. (Student's two-sided *t*-test, # = *p* = 0.06, * = *p* < 0.05).

Findings reported above indicated that Cav-1 could potentially restrict MnSOD expression via its negative regulation of Nrf2 activity in breast cancer. In a luminal breast cancer epithelial cell line devoid of Cav-1 (MCF7) we ectopically expressed Cav-1. Restoration of Cav-1 was sufficient to repress Nrf2 and, as anticipated, to suppress MnSOD expression (Figure 3A), indicating that Cav-1 indirectly regulates MnSOD expression by controlling Nrf2. Suppression of MnSOD expression by Cav-1 markedly reduced steady state levels of H_2_O_2_ (Figure 3B). This result could be explained, in part, by the detection of increased expression of antioxidant enzymes (glutathione peroxidase 1, GPx-1, and thioredoxin reductase (TXNRD3)) responsible for the clearance of H_2_O_2_ in Cav-1 reconstituted cells (Supplementary Figure S1). The reduction in steady state H_2_O_2_ levels in Cav-1 reconstituted MCF7 cells occurred in parallel to a clearly detectable reduction in the oxidation of Nrf2 negative regulator, Kelch-like ECH-associated protein 1 (Keap1), as determined by the labeling of Keap1-sulfenic acid derivatives with 5,5-Dimethylcyclohexane-1,3-dione (dimedone). Dimedone is a cyclic dione that reacts with protein-sulfenic acids to produce an adduct that can be immunologically detected and quantified (Figure 3C). Oxidation of Keap1 has been reported to result in the disassembly of the Keap1/Nrf2 complex, leading to Nrf2 nuclear translocation and activation as a transcription factor [31]. Cav-1 was found to form a ternary complex interacting directly with both Keap1 and Nrf2. As shown in Figure 3D, Cav-1 binding to Keap1 and Nrf2 was strongly suppressed by exogenous H_2_O_2_, indicating that fluctuations in the levels of H_2_O_2_ can simultaneously lead to the release of Nrf2/Keap1 from Cav-1 and the dissociation of Nrf2 from Keap1. The suppressive effect of Cav-1 on the accumulation of Nrf2 was confirmed using a NanoLuc reporter system (Nrf2-luciferase conjugated protein) by Promega (Madison, WI). We found that Cav-1 expression in MCF7 cells resulted in a reduction of Nrf2 accumulation which we attribute to the faster degradation of Nrf2 in Cav-1 reconstituted MCF7 cells (Figure 3E). Lower Nrf2 stability led to reduced MnSOD protein expression, an effect that was recapitulated either by Cav-1 reconstitution or by silencing Nrf2 (Figure 3F). These results position Nrf2 as a primary effector of MnSOD upregulation in breast cancer cells with a Cav-1 null phenotype.

### Reconstitution of Cav-1 suppresses MnSOD-dependent AMPK activation and AMPK-driven glycolysis in breast cancer epithelial cells

**Figure 4:**
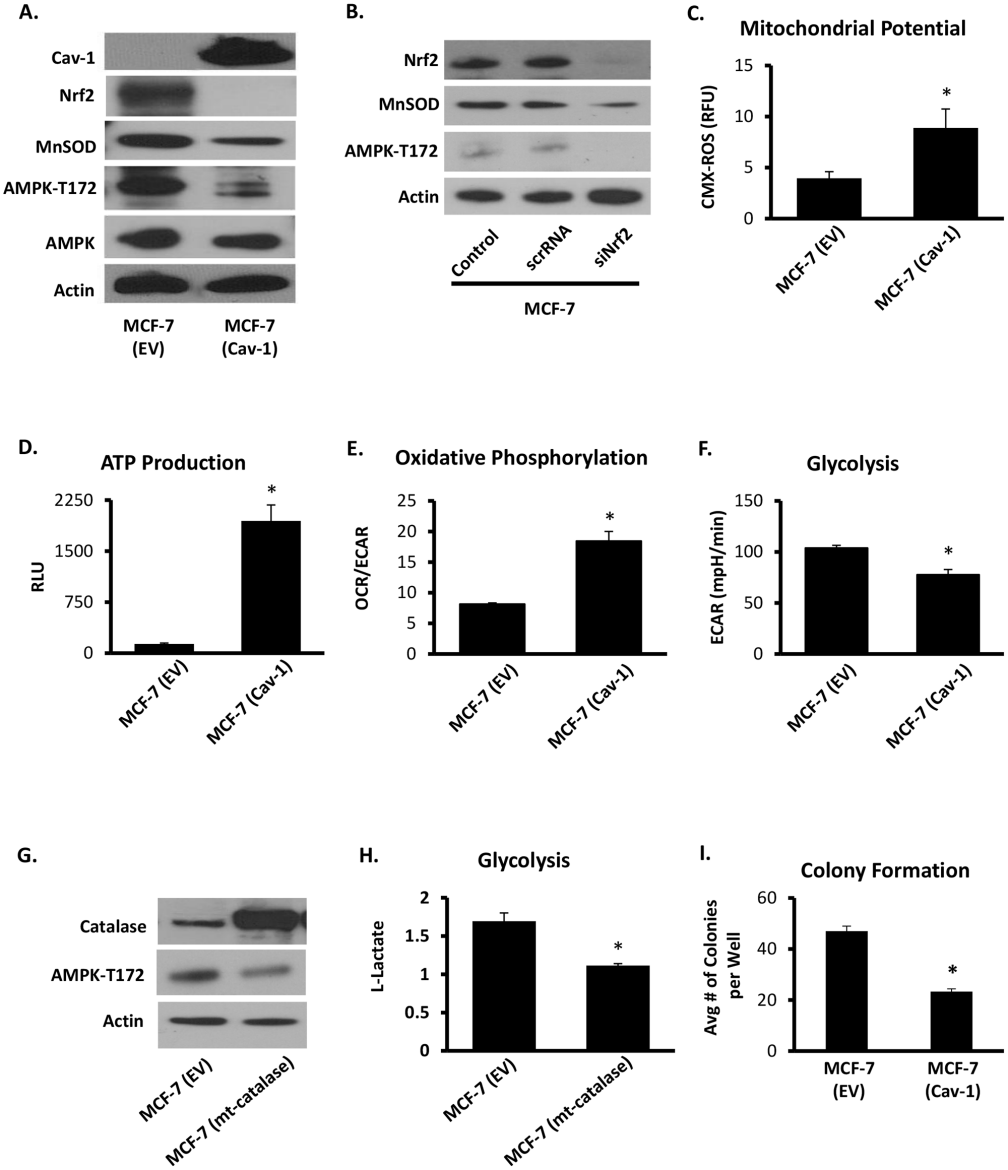
Cav-1 impedes AMPK-dependent glycolysis through inhibition of MnSOD-dependent H_2_O_2_ **A.** Western blot of MCF7 cells expressing empty vector (EV) or ectopic Cav-1 (Cav-1), in which Cav-1 expression reduces Nrf2 and MnSOD expression as well as AMPK activation as indicated by its active phosphorylation site Thr172. **B.** Nrf2 silencing in MCF7 cells (see *methods*) alone was sufficient to reduce MnSOD expression and AMPK activation. **C.** Cav-1 expression in MCF7 cells rescued mitochondrial potential as determined by CMX-ROS, in addition to restoring ATP production **D.** as measured by a luciferase-based ATP Determination Kit (Thermo Fisher Scientific). **E.** Cav-1 reconstitution in MCF7 cells increased mitochondrial respiration (OXPHOS as determined by basal OCR/ECAR), and **F.** reduced glycolytic metabolism (basal ECAR), in parallel measurement using *Seahorse XF* (Seahorse Bioscience). **G.** Transfection with mitochondria-targeted catalase (mt-catalase) was sufficient to impede AMPK activation (AMPK-T172) and **H.** reduced glycolysis as measured by L-lactate production determined by Cell-Based Glycolysis Assay Kit (Cayman). **I.** Cav-1 ectopic expression in MCF7 cells markedly reduced colony formation in soft agar, indicating a reduction of clonogenecity and anchorage-independent growth. (Student's two-sided *t*-test, * = *p* < 0.05).

Results shown in Figure 4A indicate that by promoting Nrf2 association with Keap1, Cav-1 prevents Nrf2 accumulation thereby suppressing MnSOD protein expression. Consistent with previous studies [26], one measurable effect of reducing MnSOD protein expression was the deactivation of AMPK as indicated by a reduction in phosphorylation of the AMPK activation site Thr172 (Figure 4B). Suppression of AMPK phosphorylation resulted in an increase in mitochondrial electrochemical potential (Figure 4C), an enhancement in the steady state levels of ATP (Figure 4D), and an increase in OCR/ECAR indicating a shift towards aerobic respiration (Figure 4E). The dampening of AMPK activation also resulted in inhibition of phosphofructokinase 2 (PFK2) activation, a rate limiting enzyme in the glycolytic pathway positively regulated by AMPK (Supplementary Figure S2). Consistent with these observations, we also found that glycolysis is suppressed in Cav-1 reconstituted cells (Figure 4F). The effects of Cav-1 in MCF7 cells could be reversed by the ectopic expression of MnSOD by adenoviral transfection (Supplementary Figure S3A). This result indicates that the increase in MnSOD expression that results in increased H_2_O_2_ production and the activation of glycolysis is secondary to the loss of Cav-1 expression (Supplementary Figure S3B). Mechanistically, it was also found that, consistent with previous findings [26], AMPK is activated by H_2_O_2_ originating from the mitochondria since the transfection of a mitochondria-targeted catalase (mt-catalase) that prevents the efflux of H_2_O_2_ from mitochondria, reduced both AMPK activation (Figure 4G) and glycolysis (Figure 4H). Consistent with the hypothesis that loss of Cav-1 expression promotes more aggressive cancer phenotypes, rescue of Cav-1 expression in MCF7 cells led to a marked inhibition of anchorage-independent growth as determined by a reduction in colony formation in soft agar (Figure 4I). Overall, these results indicate that Cav-1 expression suppresses MnSOD-dependent glycolysis and anoikis that characterize more aggressive cancer phenotypes.

### Cav-1 loss during malignant transformation leads to Nrf2-driven MnSOD upregulation

**Figure 5:**
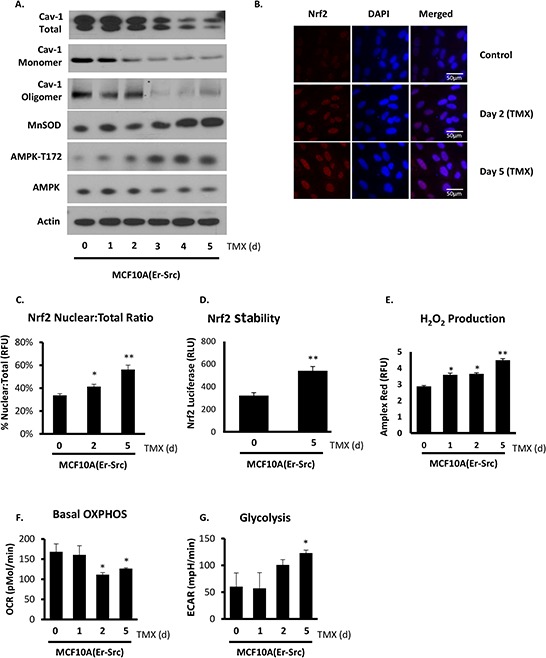
Cav-1 loss during malignant transformation is associated with increased Nrf2 activity and MnSOD upregulation that leads to AMPK-dependent glycolytic metabolism MCF10A(Er/Src) cells were used to determine metabolic changes during malignant transformation. MCF10A(Er/Src) cells were treated with 1 μg/mL tamoxifen (TMX) for the times indicated. **A.** Cav-1 is reduced early in transformation (24 and 48 h), followed by upregulation of MnSOD and AMPK activation (72 h), as determined by Western blot. Cav-1 oligomer/monomer distribution was assessed on the same non-reducing gel. **B.** Nrf2 nuclear localization is enhanced following Cav-1 loss at 48 h and is sustained following transformation. **C.** Quantification of the ratio of nuclear Nrf2 to total Nrf2 expression shows a predominant nuclear localization of Nrf2 during and following transformation. **D.** Quantification of Nrf2 stability suggests increased Nrf2 transcriptional activity in MCF10A(Er/Src) cells following transformation at 5 days post TMX induction. **E.** H_2_O_2_ production is increased as early as 24 h following TMX induction of v-Src and is significantly elevated following transformation at 96 and 120 h, consistent with the high expression of MnSOD maintained after v-Src induced transformation. **F.** OXPHOS is reduced in MCF10A(Er/Src) cells during and following transformation, and in parallel glycolysis is markedly increased following transformation **G.** (One-way ANOVA with post-hoc two-sided *t*-test, * = *p* < 0.05, ** = *p* < 0.005).

Using a model of inducible transformation, we tested the concept that loss of Cav-1 during the transition to malignancy sets in motion Nrf2 activation and through it, MnSOD-driven metabolic reprogramming towards glycolysis. Tamoxifen-induced v-Src expression in non-malignant MCF10A(Er/Src) cells led to the rapid downregulation of Cav-1 expression. Cav-1 downregulation was progressive and preceded full transformation of MCF10A(Er/Src) to a tumorigenic phenotype that has been reported to occur between 48–72 h following stimulation [32]. As shown in Figure 5A, Cav-1 levels were reduced 24 h after v-Src induction with peak suppression at 72 h. Starting at 72 h, Cav-1 loss was accentuated by the loss of oligomers (previously reported to be the functional form of Cav-1 [5]). We also measured significantly increased MnSOD expression and AMPK activation particularly after 72 h following stimulation. Cav-1 loss in transforming cells was associated with the accumulation of Nrf2 in the nucleus as early as 48 h after induction of v-Src expression (Figure 5B and 5C). This enhancement in Nrf-2 nuclear localization was consistent with an increase in its stability in transformed MCF10A(Er/Src) cells (Figure 5D). In parallel with the increase in MnSOD expression and AMPK phosphorylation, steady state H_2_O_2_ levels progressively increased during v-Src-driven transformation (Figure 5E). The increase in H_2_O_2_ levels was in parallel with the shift from oxidative to glycolytic metabolism (Figure 5F and 5G). Taken together, these results demonstrate that the identified pathway of metabolic adaptation centered on MnSOD upregulation via Nrf2 is preceded by and dependent on Cav-1 degradation.

### Cav-1 loss is associated with MnSOD overexpression and AMPK activation in an autochthonous mouse model of invasive breast carcinogenesis

**Figure 6:**
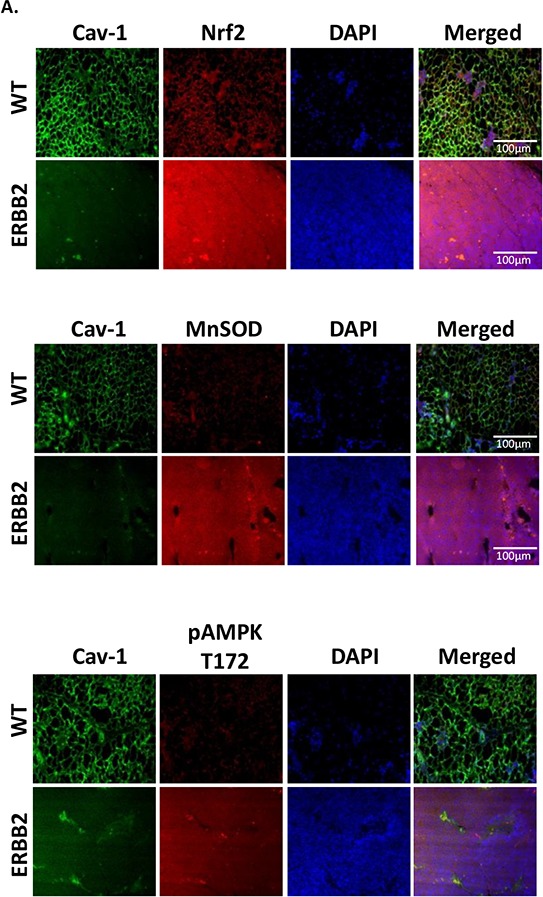
Cav-1 is repressed and MnSOD is increased in an *in vivo* model of malignancy To investigate if Cav-1 may regulate MnSOD expression in an *in vivo* model of mammary tumor development, we used the FVB-(MMTV-ERBB2) mice, which express the activated ErbB2 (*c-neu*) gene and develop spontaneous mammary tumors within 9 months [33]. We observed that, compared to control FVB mice, ERBB2 mice demonstrated a ubiquitous loss of Cav-1 that was associated with increased expression of Nrf2 and MnSOD as well as increased activation of AMPK.

Next, we determined whether Cav-1 expression is reduced in mammary tumors of genetically modified mice constitutively expressing activated c-neu (ErbB2) gene (FVB-MMTV-ErbB2 [33]). As shown in Figure 6, immunostaining of Cav-1 in tissue sections obtained from healthy mammary glands of FVB controls as compared to mammary tumors from ErbB2 mice showed generalized reduction of Cav-1 expression in all ErbB2 mouse tumors analyzed, which also displayed elevated MnSOD expression and increased AMPK (Thr172) phosphorylation.

## DISCUSSION

Our group has had a long-standing interest in the mechanistic underpinnings of the glycolytic switch in cancerous cells. Recently, we reported that the upregulation of MnSOD expression is required and sufficient for the metabolic reprogramming. Following this observation we sought to define molecular events that promote MnSOD expression in cancer. We found that Cav-1 protein levels in invasive ductal carcinoma are reduced early during tumor progression. Loss of Cav-1 expression is accentuated as tumors progress towards advanced and more aggressive stages (Figure 2). We also found that Cav-1 loss leads to an increased glycolytic activity that is imparted by MnSOD and its activation of AMPK via elevated H_2_O_2_ (Figure 5). These observations led us to hypothesize that the loss of Cav-1 is connected to the increase in MnSOD expression via Nrf2, a nuclear factor upstream of MnSOD that is scavenged by Cav-1. Here, we found that Nrf2 is, in fact, unleashed in Cav-1-deficient cells and that it indirectly enhances glycolysis in a manner dependent on the induction of MnSOD expression and MnSOD-derived H_2_O_2_. The findings of a mechanistic link between Cav-1 and MnSOD was reinforced by the analysis of existing epidemiologic data indicating that Cav-1^low^/MnSOD^high^ represents a molecular signature consistent with that of glycolytic tumors that are typically more aggressive (Table 1). In fact, the reconstitution of Cav-1 expression in invasive MCF7 ductal carcinoma cells effectively reduced MnSOD expression and steady state H_2_O_2_ levels. Dampening H_2_O_2_, resulted in the preservation of Keap-1 and of its inhibitory association with Nrf2. Surprisingly, we also found that Cav-1 binds directly to both Nrf2 and Keap1 forming a ternary complex. In the absence of Cav-1 expression, significant Keap-1 oxidation was observed (Figure 3C). As shown before, oxidized Keap-1 dissociates from Nrf2 to promote its nuclear translocation and transcriptional activity [34–36]. This finding indicates that Cav-1 loss and oxidative stress collaborate to promote the disassembly of the Nrf2/Keap1 complex thereby promoting the upregulation of MnSOD that leads to the glycolytic switch, as described previously [26]. It also supports the notion that the duration of oxidative stress can be determinant to elicit protective or pathologic functions of Nrf2. When transiently activated by spikes in H_2_O_2_ production, Nrf2 activates protective antioxidant defenses [8]; however, if persistently activated, Nrf2 leads to prolonged and marked enhancement of MnSOD expression that is sufficient to overcome antioxidant defenses and activate glycolysis, a form of metabolism permissive to malignant transformation. To further our mechanistic findings, a detailed analysis of Cav-1 and MnSOD expression was conducted using clinical samples (Figure 1). The results indicate that Cav-1 expression is progressively reduced in breast cancer according to clinical stage and histologic grade and in aggressive breast cancer subtypes.

Consistently, we found that Cav-1 is strongly downregulated during induced malignant transformation. Cav-1 downregulation happened early after the induction of transformation by v-Src expression and was followed by increased Nrf2 stability and concomitant MnSOD upregulation, the enhancement in H_2_O_2_ production, AMPK activation, and to the activation of glycolysis.

**Figure 7:**
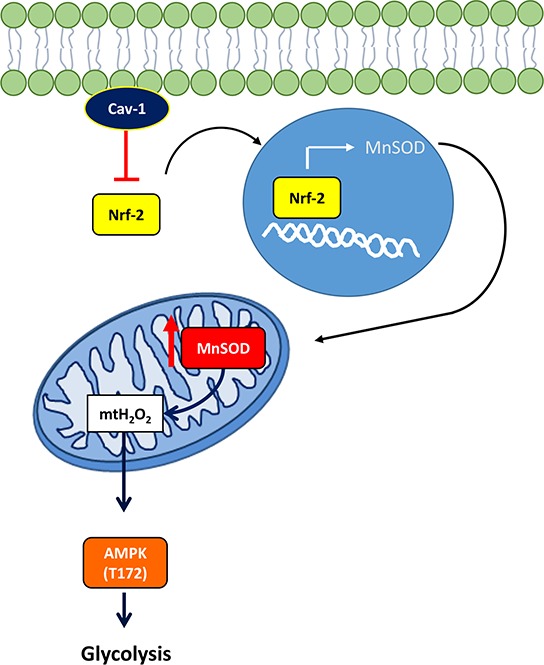
Schematic: Cav-1 prevents Nrf2 dependent upregulation of MnSOD, thereby preventing the metabolic shift to glycolysis in breast cancer Cav-1 prevents MnSOD overexpression in human breast cancer by suppression of Nrf2 transcriptional activity, thus reducing excess mtH_2_O_2_ egression from mitochondria and inhibiting downstream signaling events that bolster glycolysis.

Together, our observations can be explained by a model in which the degradation of Cav-1 unleashes Nrf2 activity and the upregulation of MnSOD (see Figure 7). Upregulated MnSOD is a source of H_2_O_2_ that activates AMPK thereby inducing the glycolytic switch. In this regard, this study provides epidemiologic and mechanistic evidence indicating that the loss of Cav-1 expression promotes a form of metabolism that is permissive to breast cancer progression and, therefore, can negatively impact patient outcome. Based on the results reported here, we postulate that the levels of Cav-1 and MnSOD expression are determinant to metabolic changes that support tumor progression and that the Cav-1^low^/MnSOD^high^ phenotype indicates a subgroup of aggressive types of breast cancer with poorer prognosis.

## MATERIALS AND METHODS

### Cell culture

Cell cultures of MCF7 cells stably expressing a myc-tagged empty vector (EV) or Cav-1 wild-type (Cav-1) were generated as described previously [37]. The cells were cultured in IMEM medium (Cellgro, Manassas, VA) supplemented with 10% fetal bovine serum (Atlanta Biologicals, Lawrenceville, GA) and 1% penicillin/streptomycin (Thermo Fisher Scientific Life Sciences, Grand Island NY). MCF10A(Er/Src) cells were a generous gift from Dr. Kevin Struhl (Harvard University). They were cultured as described in [38].

### Mitochondrial-targeted catalase transfection

Mitochondrial targeted catalase (Ad5-CMV-MT-catalase; mito-catalase) [39] was a generous gift from Dr. J. Andres Melendez (State University of New York at Albany). MCF7 cells were transfected with mito-catalase adenovirus by adding 1 × 10^6^ infectious units to 80% confluent dishes, agitated every 15 min for 1 h at 37°C, and then serum free media was added to prevent drying. After 6 h, 2 mL of complete media was added, and then changed to fresh media after 24 h.

### Silencing of Nrf2 using siRNA

To silence Nrf2 in MCF7 cells, siRNA specific for Nrf2 (Santa Cruz, Dallas, TX) was used. Briefly, cells were allowed to grow to 80% confluence and then treated with Nrf2-siRNA or scrambled-RNA using RNAiMAX (Thermo Fisher Scientific, Carlsbad, CA) in Opti-Mem (Thermo Fisher Scientific) according to the manufacturer's standard protocol. After 6 h media was increased to prevent drying, and at 24 h media was changed to complete media. Lysates for mito-catalase and Nrf2-siRNA were obtained at 72 h after transfection.

### Western Blot Analysis

Protein lysates were separated on 4–12% Bis-Tris gels and transferred to nitrocellulose membranes. The membranes were blocked for 60 min at room temperature in 5% milk in TBS-T (0.05% Tween-20, pH 7.4), washed with TBS-T and then incubated with primary antibody [rabbit anti-Cav-1 (Abcam, Cambridge, MA), mouse anti-Cav-1 (BD Technologies, Research Triangle Park, NC), rabbit anti-β-actin (Cell Signaling Technologies, Boston, MA), rabbit anti-MnSOD(Abcam), rabbit anti-AMPK pThr-172 (Abcam), rabbit anti-Nrf2(Santa Cruz), mouse anti-Keap1 (Abcam), rabbit anti-Cysteine Sulfenic Acid (EMD Millipore, Billerica, MA)] in TBS-T overnight at 4°C. Antibodies were diluted 1:1000 unless stated otherwise. After 3 washes, membranes were incubated with the infrared secondary antibody to anti-rabbit/mouse, 1:5,000 in TBS-T and incubated for 2 hours at room temperature. After 3 washes, protein signals were analyzed by Odyssey CLx Infrared Imaging System (LI-COR Biosciences, Lincoln, NE).

### Detection of cysteine oxidation and Keap1/Nrf2/Cav-1 complex stability by immunoprecipitation

Whole cell protein lysates were prepared from 90% confluent cell cultures in 100 mm culture dishes (Sigma, St. Louis, MO). Briefly, cell pellets were resuspended in 100 mM 5,5-dimethylcyclohexane-1,3-dione (Dimedone, Sigma) and incubated at 37°C for 60 min under agitation. After washing with PBS, pellets were lysed in RIPA buffer (Cell Signaling) and then sonicated for 15 seconds using Sonic Dismembrator (ThermoFisher). Following sonication, supernatant was moved to a new tube and samples were incubated with 2 μg/mL primary antibody [mouse anti-Keap1, 1:100 (Abcam)] for 24 h at 4°C. Magnetic beads [Dynabeads M-280 anti-mouse IgG (Thermo Fisher Scientific)] was then added and samples were incubated for 3 hours at 4°C. After 3 washes, samples were analyzed by Western blotting as described above.

For co-immunoprecipitation of Cav-1/Keap1/Nrf2 complex, a direct immunoprecipitation method was used. Briefly, magnetic beads [Dynabeads M-280 anti-mouse or anti-rabbit IgG (Thermo Fisher Scientific)] were incubated with primary antibody [anti-rabbit Nrf2 1:100 (Santa Cruz) or anti-mouse Keap1 1:100 (Abcam)]) for 6 h at 4°C. Beads were then washed to remove unbound primary antibody. Following treatment (400 μM H_2_O_2_, 1 h), cell lysates were added to the Dynabead/Antibody cocktail and incubated at 4°C overnight. Samples were washed twice with PBS and then analyzed by Western blot.

### Nrf2 luciferase assay

Cells were grown to 80% confluence in 6 well dishes, and then co-transfected with pNLF1-NRF2[CMV/neo] and pKeap1 according to manufacturer's protocol (Promega, Madison, WI) using Continuum Transfection Reagent (Gemini Bio-Products, Sacramento, CA). After 24 hours, cells were moved to 96-well white walled plates and incubated for an additional 48 hours. To determine Nrf2 reporter stability, Nano-Glo Luciferase Assay System (Promega) was used and luminescence was measured on a SpectraMax M5 spectrophotometer (Molecular Devices, Sunnyvale, CA). Measurements were then normalized to protein concentration measured through bicinchoninic acid (BCA) protein assay reagent (Thermo Fisher Scientific, Waltham, MA).

### Amplex Red assay

H_2_O_2_ production in cell culture was measured by Amplex Red Hydrogen Peroxide/Peroxidase Assay Kit (Thermo Fisher Scientific). Cells were grown to 80% confluence in a 6-well plate, washed twice with PBS and then incubated with Amplex Red reaction buffer for 30 min at 37°C. Amplex Red reaction buffer was then moved to a 96-well white walled plate and fluorescence was read at 560_EX_/590_EM_ on a SpectraMax M5 spectrophotometer (Molecular Devices) and normalized to protein concentration determined by BCA (Thermo Fisher Scientific).

### ATP assay

Cells were grown in a 96-well white walled plate in standard media to 80% confluence. Media was replaced with glucose free media supplemented with galactose. Steady state ATP levels were measured using the luciferase based ATP determination kit (Thermo Fisher Scientific). ATP concentration in cell lysates was measured by luminescence on a SpectraMax M5 spectrophotometer (Molecular Devices) and normalized to protein concentration by BCA (Thermo Fisher Scientific).

### Glycolysis assay

Cells were grown in 96-well clear plates in standard media to 80% confluence and Glycolysis Cell-Based Assay Kit (Cayman Chemical, Ann Arbor, MI) was performed according to manufacturer's instruction. Briefly, media was replaced with serum free media and incubated for 6 h at 37°C. Supernatant (media) was moved to a 96-well plate and glycolysis reaction mix was added. Glycolysis was determined by L-lactate release into media which was read at 490nm absorbance on a SpectraMax M5 spectrophotometer (Molecular Devices) and then normalized to BCA (Thermo Fisher Scientific).

### Mitochondrial respiration assay

Cells were grown in *Seahorse Bioscience* (North Billerica, MA) plates as a monolayer at 80% confluence. Cells were then washed and left in *Seahorse* bicarbonate free media for 2 hours at 37°C, then analyzed on the *Seahorse* Extracellular Flux (XFe24) Analyzer. The XFe24 Analyzer measures live cell oxygen consumption rate (OCR) and extracellular acidification rate (ECAR) in triplicate within intervals of 15 min. Basal OCR is used as an indicator of mitochondrial respiration, and basal ECAR is used as an indicator of glycolysis. All measurements were standardized to cell count at seeding (4 × 10^4^ cells) and additionally normalized by BCA protein concentration (Thermo Fisher Scientific).

### Confocal microscopy

Cells were plated and grown on confocal dishes (MatTek, Ashland, MA) in standard media to appropriate (60–80%) confluence. Cells were then washed three times for 3 min with 1% PBS and fixated in 4% paraformaldehyde (Sigma) for 10 min. Cells were washed three times in 1% PBS for 3 min and permeabilized in 100% methanol for 15 min. Cells were washed in 1% PBS three times for 3 min each and then blocked using 5% bovine serum albumin (BSA, Sigma) for 60 min. Cells were then washed in 1% TBS-T three times for 3 min and incubated in primary antibodies [rabbit anti-Cav-1–1:100 (Abcam), mouse anti-Cav-1–1:100 (BD Technologies), rabbit anti-MnSOD- 1:100 (Abcam), goat anti-MnSOD-1:100 (Santa Cruz), rabbit anti-Nrf2–1:100 (Santa Cruz)] in 1% BSA in 1% TBS-T overnight at 4°C in a humid chamber. Cells were washed three times in 1% TBS-T for 3 min and incubated with secondary fluorescent antibodies [rabbit Alexfluor-568, mouse Alexafluor-488 and goat Alexafluor-647 (Thermo Fisher Scientific)] for 2 h at room temperature in a dark, humid chamber. Cells were then washed three times in 1% TBS-T for 3 min and incubated with 50 μM DAPI (Thermo Fisher Scientific) for 30 min. Cells were washed twice in 1% TBS-T for 3 min and once in PBS for 5 min and then imaged using LSM-510META (Carl Zeiss Microscopy, Thornwood, NY).

### Fluorescent immunohistochemistry

Antigen retrieval was done using 10 mM Sodium Citrate buffer at 20 psi for 5 min in a Decloaking Chamber electric pressure cooker (Biocare Medical, Walnut Creek, CA). Slides were blocked with 10% FBS for 45 min and incubated with primary antibody [rabbit anti-Cav-1–1:100 (Abcam), mouse anti-Cav-1–1:100 (BD Technologies), rabbit anti-MnSOD-1:100 (Abcam), goat anti-MnSOD-1:100 (Santa Cruz), rabbit anti-Nrf2–1:100 (Santa Cruz)] overnight at 4°C in a humid chamber. Non-immune IgG was used for negative control. After rinsing in TBS-T, sections were incubated with secondary antibody [rabbit Alexfluor-568 and mouse Alexafluor-488 (Thermo Fisher Scientific)] for 2 h at room temperature in a dark, humid chamber. After incubating with 50 μM DAPI, slides were mounted with FluoroMount Aqueous Solution (Sigma) and examined on an Apotome fluorescen microscope (Zeiss).

### Corrected total fluorescence

Corrected total fluorescence was calculated as described previously [40]. Briefly, post-processing proceeded in ImageJ (National Institute of Health, Bethesda, MD) of each channel obtained during immunofluorescent acquisition of histological samples. Integrated density was used to measure total intensity (relative fluorescent units, RFU) within each selection of each channel, corrected against background mean grey value and area of measurement, and compiled across samples. Relative fluorescent units (RFU) as determined by corrected total fluorescence was calculated as follows: Integrated density of selection – (area of selection x mean background integrated density). Three background samples were taken per selection to assure proper calibration. Statistical analysis was performed as described below.

### Soft agar assay

To evaluate clonogenecity and anchorage-independent growth, soft agar assay was performed. Briefly, 2 × 10^5^ cells were seeded in 12 well dishes in 0.4% soft agar in native media on top of a layer of 0.8% soft agar (2X DMEM, 20%FBS, 1% Pen/Strep). Colonies were allowed to grow for 4 weeks, and then stained using Trypan Blue (Thermo Fisher Scientific). Colonies were imaged by EVOS Cell Imaging Systems (Thermo Fisher Scientific) and counted using ImageJ. Colonies were scored if at least 5 cells were present within one cluster.

### Epidemiology

Data was obtained from the Oncomine® database (Compendia Bioscience, Ann Arbor, MI) using the Sorlie-Breast [27], Curtis-Breast [28] and Kao-Breast [29] datasets. Data and graphs were arranged in Microsoft Excel (Microsoft, Redmond, WA) and statistics were conducted using IBM SPSS Statistics 20 (IBM Corporation, Armonk, NY). For odds ratio (OR) determination, binary/binomial logistic regression was performed using SPSS.

### Statistical analysis

Statistical analysis was performed with GraphPad InStat. One-way ANOVA was performed with post-hoc Student-Newman-Keuls comparisons. A value of *P* < 0.05 was considered significant whereas *a* value of *P* < 0.01 was considered highly significant.

## SUPPLEMENTARY FIGURES



## References

[1] Lisanti MP, Scherer PE, Vidugiriene J, Tang Z, Hermanowski-Vosatka A, Tu YH, Cook RF, Sargiacomo M (1994). Characterization of caveolin-rich membrane domains isolated from an endothelial-rich source: implications for human disease. J Cell Biol.

[2] Rothberg KG, Heuser JE, Donzell WC, Ying YS, Glenney JR, Anderson RG (1992). Caveolin, a protein component of caveolae membrane coats. Cell.

[3] Lisanti MP, Scherer PE, Tang Z, Sargiacomo M (1994). Caveolae, caveolin and caveolin-rich membrane domains: a signalling hypothesis. Trends Cell Biol.

[4] Schnitzer JE, Liu J, Oh P (1995). Endothelial caveolae have the molecular transport machinery for vesicle budding, docking, and fusion including VAMP, NSF, SNAP, annexins, and GTPases. J Biol Chem.

[5] Sargiacomo M, Scherer PE, Tang Z, Kubler E, Song KS, Sanders MC, Lisanti MP (1995). Oligomeric structure of caveolin: implications for caveolae membrane organization. Proc Natl Acad Sci U S A.

[6] Liu J, Oh P, Horner T, Rogers RA, Schnitzer JE (1997). Organized endothelial cell surface signal transduction in caveolae distinct from glycosylphosphatidylinositol-anchored protein microdomains. J Biol Chem.

[7] Lu ML, Schneider MC, Zheng Y, Zhang X, Richie JP (2001). Caveolin-1 interacts with androgen receptor. A positive modulator of androgen receptor mediated transactivation. J Biol Chem.

[8] Volonte D, Liu Z, Musille PM, Stoppani E, Wakabayashi N, Di YP, Lisanti MP, Kensler TW, Galbiati F (2013). Inhibition of nuclear factor-erythroid 2-related factor (Nrf2) by caveolin-1 promotes stress-induced premature senescence. Molecular biology of the cell.

[9] Schlegel A, Wang C, Katzenellenbogen BS, Pestell RG, Lisanti MP (1999). Caveolin-1 potentiates estrogen receptor alpha (ERalpha) signaling. caveolin-1 drives ligand-independent nuclear translocation and activation of ERalpha. J Biol Chem.

[10] Brouet A, Sonveaux P, Dessy C, Moniotte S, Balligand JL, Feron O (2001). Hsp90 and caveolin are key targets for the proangiogenic nitric oxide-mediated effects of statins. Circ Res.

[11] Mao M, Varadarajan S, Fukai T, Bakhshi FR, Chernaya O, Dudley SC, Minshall RD, Bonini MG (2014). Nitroglycerin tolerance in caveolin-1 deficient mice. PLoS One.

[12] Lim EJ, Smart EJ, Toborek M, Hennig B (2007). The role of caveolin-1 in PCB77-induced eNOS phosphorylation in human-derived endothelial cells. Am J Physiol Heart Circ Physiol.

[13] Majkova Z, Toborek M, Hennig B (2010). The role of caveolae in endothelial cell dysfunction with a focus on nutrition and environmental toxicants. J Cell Mol Med.

[14] Isshiki M, Ando J, Yamamoto K, Fujita T, Ying Y, Anderson RG (2002). Sites of Ca(2+) wave initiation move with caveolae to the trailing edge of migrating cells. J Cell Sci.

[15] Grande-Garcia A, Echarri A, de Rooij J, Alderson NB, Waterman-Storer CM, Valdivielso JM, del Pozo MA (2007). Caveolin-1 regulates cell polarization and directional migration through Src kinase and Rho GTPases. J Cell Biol.

[16] Bakhshi FR, Mao M, Shajahan AN, Piegeler T, Chen Z, Chernaya O, Sharma T, Elliott WM, Szulcek R, Bogaard HJ, Comhair S, Erzurum S, van Nieuw Amerongen GP (2013). Nitrosation-dependent caveolin 1 phosphorylation, ubiquitination, and degradation and its association with idiopathic pulmonary arterial hypertension. Pulm Circ.

[17] Yang KC, Rutledge CA, Mao M, Bakhshi FR, Xie A, Liu H, Bonini MG, Patel HH, Minshall RD, Dudley SC (2014). Caveolin-1 modulates cardiac gap junction homeostasis and arrhythmogenecity by regulating cSrc tyrosine kinase. Circ Arrhythm Electrophysiol.

[18] Bitar MS, Abdel-Halim SM, Al-Mulla F (2013). Caveolin-1/PTRF upregulation constitutes a mechanism for mediating p53-induced cellular senescence: implications for evidence-based therapy of delayed wound healing in diabetes. Am J Physiol Endocrinol Metab.

[19] Pavlides S, Tsirigos A, Vera I, Flomenberg N, Frank PG, Casimiro MC, Wang C, Fortina P, Addya S, Pestell RG, Martinez-Outschoorn UE, Sotgia F, Lisanti MP (2010). Loss of stromal caveolin-1 leads to oxidative stress, mimics hypoxia and drives inflammation in the tumor microenvironment, conferring the “reverse Warburg effect“: a transcriptional informatics analysis with validation. Cell Cycle.

[20] Chanvorachote P, Chunhacha P (2013). Caveolin-1 regulates endothelial adhesion of lung cancer cells via reactive oxygen species-dependent mechanism. PLoS One.

[21] Simpkins SA, Hanby AM, Holliday DL, Speirs V (2012). Clinical and functional significance of loss of caveolin-1 expression in breast cancer-associated fibroblasts. J Pathol.

[22] Pavlides S, Whitaker-Menezes D, Castello-Cros R, Flomenberg N, Witkiewicz AK, Frank PG, Casimiro MC, Wang C, Fortina P, Addya S, Pestell RG, Martinez-Outschoorn UE, Sotgia F (2009). The reverse Warburg effect: aerobic glycolysis in cancer associated fibroblasts and the tumor stroma. Cell Cycle.

[23] Cerezo A, Guadamillas MC, Goetz JG, Sanchez-Perales S, Klein E, Assoian RK, del Pozo MA (2009). The absence of caveolin-1 increases proliferation and anchorage- independent growth by a Rac-dependent, Erk-independent mechanism. Mol Cell Biol.

[24] Hulit J, Bash T, Fu M, Galbiati F, Albanese C, Sage DR, Schlegel A, Zhurinsky J, Shtutman M, Ben-Ze'ev A, Lisanti MP, Pestell RG (2000). The cyclin D1 gene is transcriptionally repressed by caveolin-1. J Biol Chem.

[25] Burgermeister E, Friedrich T, Hitkova I, Regel I, Einwachter H, Zimmermann W, Rocken C, Perren A, Wright MB, Schmid RM, Seger R, Ebert MP (2011). The Ras inhibitors caveolin-1 and docking protein 1 activate peroxisome proliferator-activated receptor gamma through spatial relocalization at helix 7 of its ligand-binding domain. Mol Cell Biol.

[26] Hart PC, Mao M., Abreu A.L., Ansenberger-Fricano K., Ekou D.N., Ganini D., Kajdacsy-Balla A., Diamond A.M., Minshall R.D., Consolaro M.E., Santos J.H., Bonini M.G. (2015). MnSOD upregulation sustains the Warburg effect via mitochondrial ROS and AMPK-dependent signalling in cancer. Nat Commun.

[27] Sorlie T, Perou CM, Tibshirani R, Aas T, Geisler S, Johnsen H, Hastie T, Eisen MB, van de Rijn M, Jeffrey SS, Thorsen T, Quist H, Matese JC (2001). Gene expression patterns of breast carcinomas distinguish tumor subclasses with clinical implications. Proc Natl Acad Sci U S A.

[28] Curtis C, Shah SP, Chin SF, Turashvili G, Rueda OM, Dunning MJ, Speed D, Lynch AG, Samarajiwa S, Yuan Y, Graf S, Ha G, Haffari G (2012). The genomic and transcriptomic architecture of 2,000 breast tumours reveals novel subgroups. Nature.

[29] Kao KJ, Chang KM, Hsu HC, Huang AT (2011). Correlation of microarray-based breast cancer molecular subtypes and clinical outcomes: implications for treatment optimization. BMC Cancer.

[30] Li W, Liu H, Zhou JS, Cao JF, Zhou XB, Choi AM, Chen ZH, Shen HH (2012). Caveolin-1 inhibits expression of antioxidant enzymes through direct interaction with nuclear erythroid 2 p45-related factor-2 (Nrf2). The Journal of biological chemistry.

[31] Kansanen E, Kuosmanen SM, Leinonen H, Levonen AL (2013). The Keap1-Nrf2 pathway: Mechanisms of activation and dysregulation in cancer. Redox Biol.

[32] Iliopoulos D, Hirsch HA, Struhl K (2009). An epigenetic switch involving NF-kappaB, Lin28, Let-7 MicroRNA, and IL6 links inflammation to cell transformation. Cell.

[33] Peng M, Ball-Kell SM, Franks RR, Xie H, Tyner AL (2013). Protein tyrosine kinase 6 regulates mammary gland tumorigenesis in mouse models. Oncogenesis.

[34] Itoh K, Wakabayashi N, Katoh Y, Ishii T, Igarashi K, Engel JD, Yamamoto M (1999). Keap1 represses nuclear activation of antioxidant responsive elements by Nrf2 through binding to the amino-terminal Neh2 domain. Genes Dev.

[35] Dhakshinamoorthy S, Jaiswal AK (2001). Functional characterization and role of INrf2 in antioxidant response element-mediated expression and antioxidant induction of NAD(P)H:quinone oxidoreductase1 gene. Oncogene.

[36] Fourquet S, Guerois R, Biard D, Toledano MB (2010). Activation of NRF2 by nitrosative agents and H2O2 involves KEAP1 disulfide formation. J Biol Chem.

[37] Shajahan AN, Dobbin ZC, Hickman FE, Dakshanamurthy S, Clarke R (2012). Tyrosine-phosphorylated caveolin-1 (Tyr-14) increases sensitivity to paclitaxel by inhibiting BCL2 and BCLxL proteins via c-Jun N-terminal kinase (JNK). J Biol Chem.

[38] Hirsch HA, Iliopoulos D, Joshi A, Zhang Y, Jaeger SA, Bulyk M, Tsichlis PN, Shirley Liu X, Struhl K (2010). A transcriptional signature and common gene networks link cancer with lipid metabolism and diverse human diseases. Cancer Cell.

[39] Rodriguez AM, Carrico PM, Mazurkiewicz JE, Melendez JA (2000). Mitochondrial or cytosolic catalase reverses the MnSOD-dependent inhibition of proliferation by enhancing respiratory chain activity, net ATP production, and decreasing the steady state levels of H(2)O(2). Free Radic Biol Med.

[40] Burgess A, Vigneron S, Brioudes E, Labbe JC, Lorca T, Castro A (2010). Loss of human Greatwall results in G2 arrest and multiple mitotic defects due to deregulation of the cyclin B-Cdc2/PP2A balance. Proc Natl Acad Sci U S A.

