# Supplementary material for: Caveolin-1 regulates cancer cell metabolism via scavenging Nrf2 and suppressing MnSOD-driven glycolysis
Source: Oncotarget. 2015 Oct 26;7(1):308–22. doi: 10.18632/oncotarget.5687 (PMC4808000; doi:10.18632/oncotarget.5687)
Supplement: Supplementary file 1 [file oncotarget-07-0308-s001.pdf]

SUPPLEMENTARY FIGURES

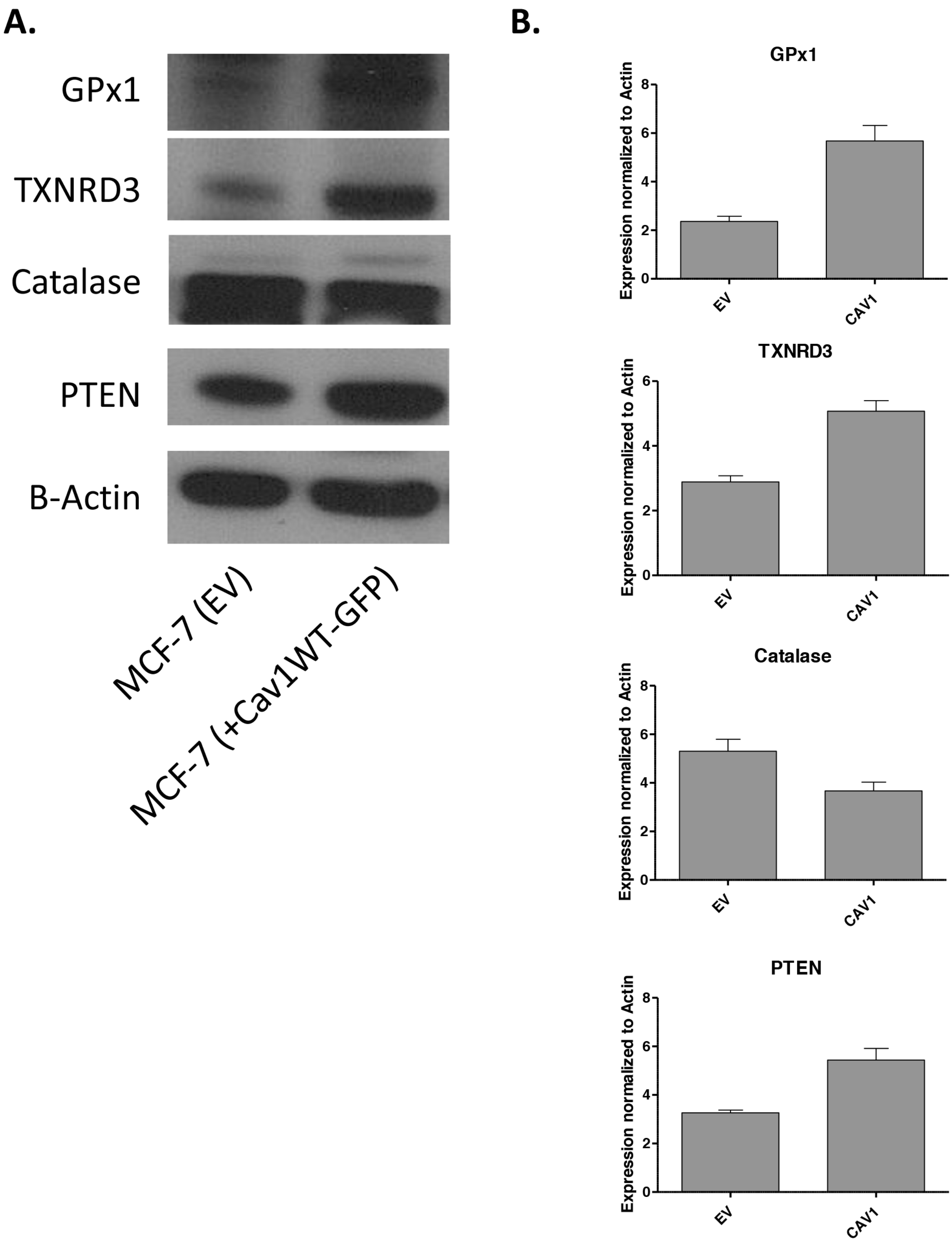

Supplementary Figure S1: Cav-1 expression enhances mitochondrial peroxidases and stabilizes the tumor suppressor PTEN. A. MCF7 cells stably expressing either empty vector or Cav-1 wild-type were analyzed by western blot, indicating an increase in glutathione peroxidase (GPx1), thioredoxin reductase (TXNRD3) and catalase. Additionally, Cav-1 expression in MCF7 cells could rescue PTEN expression, a notable tumor suppressor gene. Densitometry is presented in panel **B.**

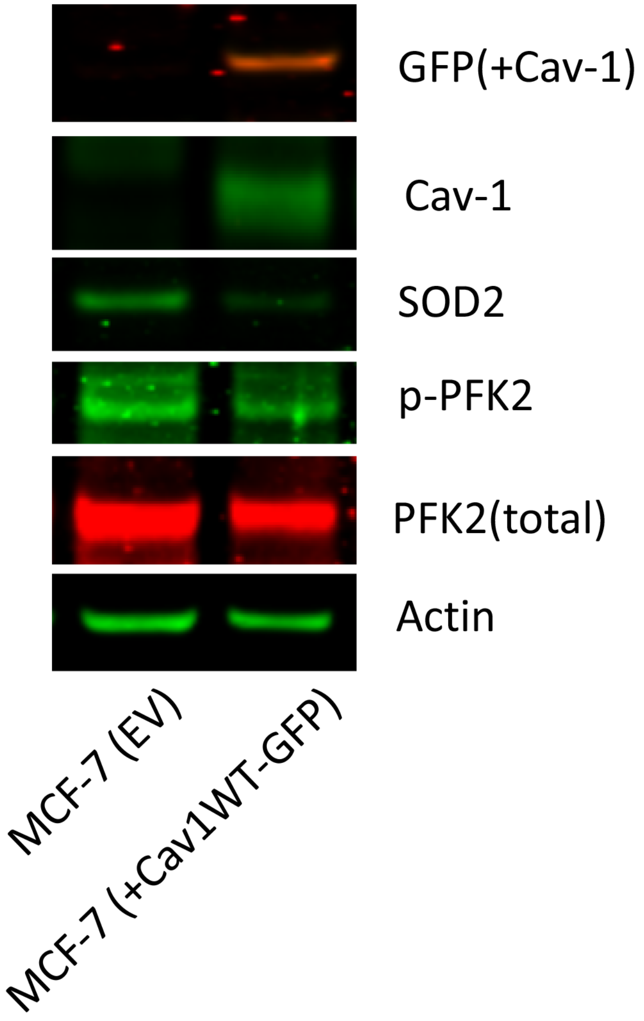

Supplementary Figure S2: Cav-1 expression reduces phosphofructokinase phosphorylation in MCF7 cells. A. To confirm the mitigation of AMPK phosphorylation and glycolysis by Cav-1, phosphorylation of phosphofructokinase 2 (PFK2), the rate limiting enzyme downstream of AMPK was analyzed by Western blot. Cav-1 consistently reduced MnSOD as well as both phosphorylated and total PFK2.

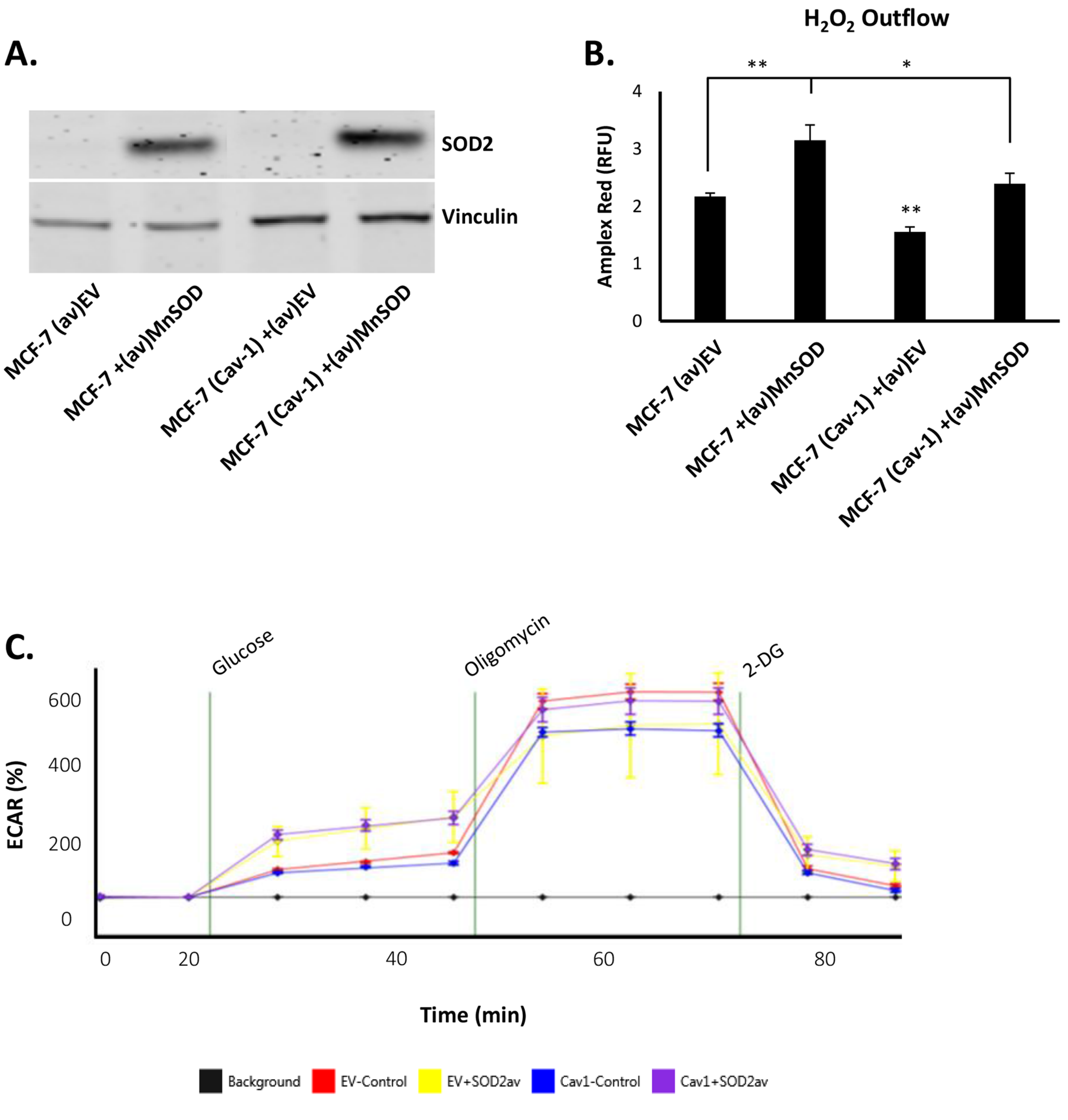

Supplementary Figure S3: MnSOD reconstitution in Cav-1 competent cells restores glycolytic metabolism. To assess that MnSOD downstream of Cav-1 was critical for the shift to glycolytic metabolism, MCF7 cells expressing either empty vector or Cav-1 wild-type were infected with MnSOD adenovirus. Panel **A.** confirms the comparable infection efficiency in both EV and Cav-1 cells; however, the degree of expression made it impossible to detect endogenous MnSOD by Western blot. **B.** Overexpression of MnSOD in either cell line was sufficient to increase the production of its product, H_2O_2. Addition of MnSOD in Cav-1 competent cells restored H_2O_2 production comparable to that of the control MCF7 cells, and further increased H_2O_2 in cells devoid of Cav-1. **C.** Seahorse electron flux assay revealed that MnSOD upregulation in both empty vector and Cav-1 reconstituted MCF7 cells enhanced glycolytic rate following glucose treatment and enhanced glycolytic capacity only in Cav-1 competent cells as determined by increased ECAR following oligomycin exposure. Both glycolytic rate and capacity were reduced in Cav-1 reconstituted cells compared to controls, consistent with our earlier findings that Cav-1 reduces glycolytic metabolism in MCF7 cells.
